# *Oreocharis fulvovillosa* (Gesneriaceae), a new species from western Yunnan, China

**DOI:** 10.3897/phytokeys.276.182136

**Published:** 2026-06-08

**Authors:** Yang Wang, Hong Jiang, Bo Xu, Meng-Qi Han

**Affiliations:** 1 Mountain Ecological Restoration and Biodiversity Conservation Key Laboratory of Sichuan Province, Chengdu Institute of Biology, Chinese Academy of Sciences, Chengdu 610213, China Kunming Institute of Botany, Chinese Academy of Sciences Kunming China; 2 Yunnan Key Laboratory of Biodiversity of Gaoligong Mountain, Yunnan Academy of Forestry and Grassland, Kunming, Yunnan, China Chengdu Institute of Biology, Chinese Academy of Sciences Chengdu China; 3 State Key Laboratory of Plant Diversity and Specialty Crops, Kunming Institute of Botany, Chinese Academy of Sciences, Kunming, Yunnan, China Yunnan Key Laboratory of Biodiversity of Gaoligong Mountain, Yunnan Academy of Forestry and Grassland Kunming China

**Keywords:** Flora of Yunnan, karst, yellow flowers

## Abstract

*Oreocharis fulvovillosa*, a new species of Gesneriaceae, is described and illustrated. This new species has only been discovered on the rock walls of an isolated karst hill at its type locality in Mang City, western Yunnan, China.

## Introduction

*Oreocharis*Bentham (1876) belongs to the Gesneriaceae subfam. Didymocarpoideae tribe Trichosporeae subtribe Didymocarpinae (Weber et al. 2013). After recent redefinition and the publication of numerous new taxa, the number of species in this genus now exceeds 160 (Xie et al. 2025; GRC 2026). *Oreocharis* is widely distributed in tropical and subtropical regions of East Asia, with most species living on steep rock cliffs of mountains based on siliceous substrates (Li 1996; Li and Wang 2005; Chen et al. 2018; Kong et al. 2022; Ling et al. 2022).

The isolated karst landform patches in western Yunnan, characterised by their thin soil layers and rugged terrain, often retain primary subtropical broad-leaved forest ecosystems. The cliff faces beneath these forests offer unique ecological niches for endemic species. Further investigation, research and conservation efforts are needed for such isolated karst landform areas that harbour unique ecosystems but are not yet included within nature reserve boundaries.

In 2019, one of the authors (HJ) encountered an unidentified species of *Oreocharis* during an orchid survey in Dehong County, Yunnan Province. During a revisit in August 2025, morphological variation within the population was documented and adjacent suitable habitats were surveyed, but no additional populations were found. The plants are perennial, epipetric herbs with a basal rosette of leaves and bear axillary cymose inflorescences. The flowers are zygomorphic, with a tubular corolla and four fertile stamens included within the corolla; the filaments are sharply bent near the apex, and the anthers are coherent in pairs. These characters, together with the presence of a single staminode and a ring-like disc, are consistent with the circumscription of *Oreocharis* before its redefinition in 2011 (Möller et al. 2011), which includes several formerly recognised genera, such as *Ancylostemon*Craib (1919). The combination of tetrandrous flowers and yellow corollas suggests an affinity with a morphologically coherent group within *Oreocharis* (Möller et al. 2011; Chen et al. 2014, 2020; Jin et al. 2021). Recent phylogenetic studies based on extensive sampling (e.g. Xie et al. 2025; Xiong et al. 2025; Shi et al. 2026) have further clarified species relationships within this group and demonstrated that morphological characters, such as corolla form, indumentum and stamen structure, are informative for species delimitation. Based on these morphological features and comparisons with herbarium specimens and the relevant literature, the Dehong population is assigned to *Oreocharis*.

Morphologically, the new species is characterised by yellow corollas and bullate leaf blades. Within *Oreocharis*, only a limited number of species share this combination of characters. Among these, it most closely resembles *Oreocharis bullata* in vegetative morphology and *O. fulva* in corolla form (Wang 1992; Möller et al. 2011; Chen et al. 2020; Xie et al. 2025). It also shows some similarity to *O. hekouensis* in general leaf shape. However, detailed morphological comparison of these species (Table 1), together with examination of the relevant literature and herbarium specimens, supports the recognition of the Dehong population as a distinct and previously undescribed species, which is described below.

**Table 1.** Morphological comparison among *Oreocharis fulvovillosa* sp. nov., *O. bullata*, *O. fulva*, and *O. hekouensis*.

Characters	*Oreocharis fulvovillosa*	*Oreocharis bullata*	*Oreocharis fulva*	*Oreocharis hekouensis*
Number of leaves	7–12	ca. 10	4–8	5–8
Petiole length	2–5 cm	1–3 cm	3–8 cm	1–4 cm
Leaf blade shape	Ovate to broadly ovate	Ovate to ovate-rhomboid	Broadly ovate	Ovate
Leaf margin	Crenulate	Double dentate	Serrate to crenate	Crenate
Adaxial surface	Bullate	Bullate	Not bullate	Not bullate
Calyx	5-lobed nearly to base	5-lobed from middle	5-lobed nearly to base	5-lobed nearly to base
Corolla tube	Slender tubular, ca. 1 cm long	Tubular, ca. 2.7 cm long	Tubular, ca. 2.4 cm long	Tubular, 2.4–3.0 cm long
Capsule	1–1.9 cm	3.0–4.2 cm	2.0–2.5 cm	2.5–3.8 cm

## Materials and methods

The methods employed here followed the general procedures for specimen examination and morphological comparison outlined in Wang et al. (1998) and Simpson (2019). Morphological characters of the new species were examined through observations of living plants and herbarium specimens. Observations of live plants were carried out on the field population, with approximately 20 mature individuals directly analysed and studied. Digital images of type specimens of the genus *Oreocharis*, available at JSTOR Global Plants (http://plants.jstor.org/), were thoroughly examined. Additionally, physical specimens of *Oreocharis* at CDBI, HITBC, IBK, IBSC, KUN and PE were examined, and high-resolution images of specimens, including types, from CSH, E, GH, K, MO and P were consulted. Herbarium acronyms follow Index Herbariorum (Thiers 2026, continuously updated). The pertinent taxonomic literature (Wang et al. 1998; Li and Wang 2005; Chen and Shui 2006; Cai et al. 2020; Gong et al. 2022; Hu et al. 2023; GRC 2026; Xie et al. 2025) was consulted to facilitate comparison with previously described species.

## Taxonomic treatment

### Oreocharis fulvovillosa

Taxon classificationPlantaeColeopteraCurculionidae

M.Q.Han, H.Jiang & Y.Wang sp. nov.

607662FA-B2D6-5918-8E70-6EBBB048A37E

urn:lsid:ipni.org:names:77381074-1

Fig. 1

#### Diagnosis.

The new species is most similar to *Oreocharis bullata*, sharing yellow corollas and bullate leaf blades, but differs in having crenulate leaf margins (vs. double dentate), a slender corolla tube ca. 1 cm long (vs. tubular, ca. 2.7 cm long), and shorter capsules 1–1.9 cm long (vs. 3.0–4.2 cm long). It is also similar to *Oreocharis fulva* in corolla shape and colour but differs in having bullate leaf blades (vs. not bullate) and crenulate margins (vs. serrate to crenate).

**Figure 1.**
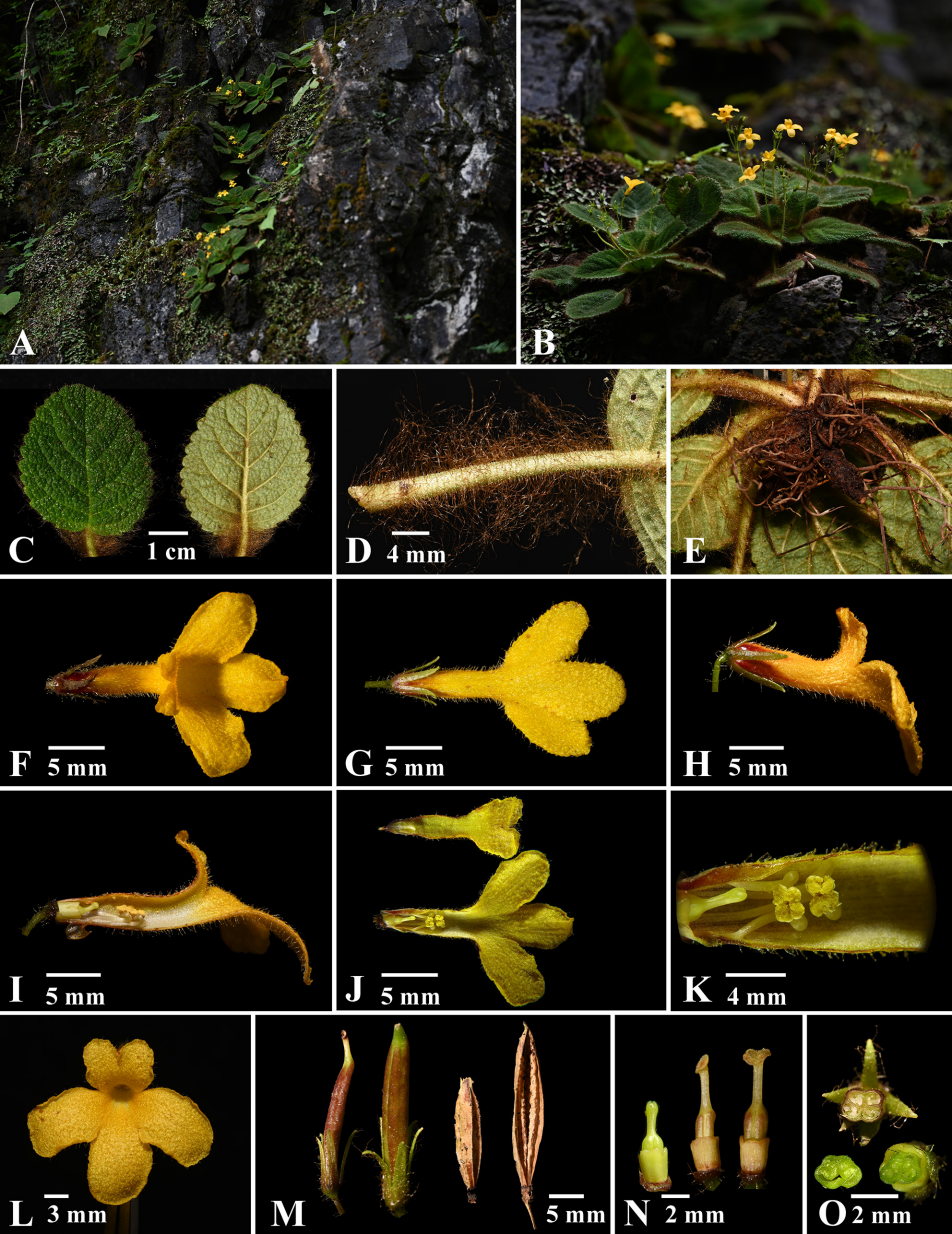
*Oreocharis fulvovillosa* sp. nov. **A**. Habitat; **B**. Plant habit in natural habitat; **C**. Adaxial (left) and abaxial (right) leaf surfaces; **D**. Petiole with long fulvous indumentum; **E**. Rhizomatous stem; **F**. Flower in adaxial view; **G**. Flower in abaxial view; **H**. Flower in side view; **I**. Flower longitudinally cut, male stage with immature pistil; **J**. Flower transversely cut, male stage with immature pistil; **K**. Immature pistil and stamens with dehisced anthers cohering in pairs; **L**. Flower in front view; **M**. Capsules at different developmental stages: two immature capsules (left) and two dehisced capsules from the previous year (right); **N**. Pistils at different developmental stages: left, at flower opening with immature stigma; middle and right, at stigma maturity; **O**. Transverse sections of ovaries, all arranged with the dorsal calyx lobe positioned upwards: upper image, ovary at stigma maturity; lower images, ovary with immature stigma, showing transverse sections of the upper part of the ovary (left) and the lower part of the ovary (right).

#### Type.

China • Yunnan Province: Dehong Dai and Jingpo Autonomous Prefecture, Mang city, 1950 m, a.s.l, on the surface of moist cliffs, 24°14'N, 98°21'E, 27 August 2025, *Mengqi Han & Yang Wang HMQ2486* (holotype: KUN [KUN1729596]; isotypes: CDIB [CDBI0309749], PE).

#### Description.

Perennial herb with very short rhizomatous stems and crowded roots. Leaves basal, arrangement spiral, 7 to 12 per plant; petioles 2–5 cm long, densely covered with long fulvous hairs; ***leaf blades*** ovate to broadly ovate, 2.5–5.5 × 3–6 cm, papery when dried, shallowly heart-shaped at bases, margins crenulate, soft hairs closely adhered to both sides, adaxial bullate; lateral veins ca. 4–6 on either side of midrib, abaxially conspicuous. Pair-flowered cymes 2–6, axillary, 2–6-flowered; peduncles 3–5 cm long, densely pilose; bracts two, opposite, lanceolate, ca. 2 mm long, sparsely pilose; pedicels ca. 2.5 cm, sparsely pilose. Calyx 5-lobed nearly to base, lobes lanceolate, nearly equal, ca. 4 mm long, outside sparsely glandular-pilose, glabrous inside. Corolla yellow, bilabiate, ca. 2 cm long; corolla tube tubular, slender, ca. 1 cm long, outside covered with glandular hairs (surface pustulose at base of hairs), glabrous inside; limb 2-lipped, both surfaces sparsely covered with glandular hairs, adaxial lip ca. 7 mm, 2-lobed to middle; abaxial lip ca. 11 mm, three-lobed to approximately one-third from the base, lobes almost equal, oblong, 6–7 × 8–9 mm. Stamens four, included; filaments white, glabrous, curved backwards near apex, adaxial ca. 12 mm long, adnate to corolla tube ca. 4 mm from base, abaxial ca. 15 mm long, adnate to corolla tube ca. 4 mm from base; anthers ca. 0.5 mm long, coherent in two pairs, elliptic, basifixed, dehiscing longitudinally, oriented upwards due to the backward curvature of the filaments; staminode one, white, glabrous, adnate to the corolla tube ca. 1.5 mm above the base, ca. 1.5 mm long. Pistil included, 8–9 mm long when stigma mature, glabrous; ovary yellowish green when unpollinated, becoming yellowish brown after fertilization, grooved, ca. 6 mm long when stigma mature, 1-loculed, placentas two, parietal, bifid, intrusive, adaxial lobes smaller; style faint yellow, 4–5 mm when stigma mature; disc ring-like with slightly undulated margin, 2–3 mm high; stigma 2-lobed, lobes ca. 1 mm long. Capsules straight relative to pedicel, brown, oblong, 10–19 × 3 mm, valves straight, dehiscing loculicidally, calyx deciduous, not caducous.

#### Phenology.

The new species has been collected in flower from August to September, and fruiting was observed during field observation in September.

#### Etymology.

The epithet refers to the fulvous (yellowish) colour of the long, soft hairs on the petioles.

#### Vernacular name.

Chinese Mandarin: dé hóng zhí bàn jù tái, (德宏直瓣苣苔).

#### Distribution, habitat, and ecology.

*Oreocharis fulvovillosa* was observed growing on moist, shady cliffs on a limestone hill, 1950 m a.s.l., in the Dehong Dai and Jingpo Autonomous Prefecture of Yunnan, China.

#### Conservation status.

*Oreocharis fulvovillosa* is a rare species with an extremely restricted distribution and a very small population size. It is currently known only from the type locality in an isolated karst area of Mang City, which lies outside any formally protected area. Targeted field surveys were conducted in the surrounding areas within a radius of approximately 1 km, but no additional subpopulations were located.

At present, the species is known from a single location, near the summit of a small limestone hill, where the plants grow on a cliff face of approximately 50 × 50 m. The extent of occurrence (EOO) cannot be meaningfully calculated from a single known location, and the area of occupancy (AOO) is estimated as 4 km^2^ based on the IUCN standard 2 × 2 km grid. The total number of mature individuals is estimated to be approximately 70.

Given its extremely limited distribution, single location and small population size, the species is potentially threatened by stochastic events and human disturbance (e.g. habitat degradation or collection). Following the IUCN Red List Categories and Criteria (IUCN 2024), *O. fulvovillosa* qualifies as Critically Endangered (CR B1ab(iii)+B2ab(iii); D). This assessment should be regarded as preliminary and subject to revision pending further field surveys in adjacent karst areas.

#### Additional specimens examined (paratypes).

China • Yunnan, Dehong Dai and Jingpo Autonomous Prefecture, Mang city, 24°14'N, 98°21'E, 1950 m, a.s.l, on the surface of moist cliffs, 5 September 2020, *Mengqi Han HMQ1823* (PE).

#### Notes.

*Oreocharis hekouensis* (Y.M.Shui & W.H.Chen) Mich.Möller & A.Weber is included in Table 1 for comparison because it shares a generally similar leaf shape with the new species. However, it can be readily distinguished from *O. fulvovillosa* by several characters. The new species has bullate leaf blades (vs. not bullate), crenulate margins (vs. crenate) and a much shorter corolla tube ca. 1 cm long (vs. 2.4–3.0 cm long). In addition, the calyx lobes are shorter, ca. 4 mm long (vs. ca. 10 mm long), the stamens are adnate to the corolla tube for 2–2.5 mm (vs. 7–8 mm), and the capsules are 1–1.9 cm long (vs. 2.5–3.8 cm long).

## Supplementary Material

XML Treatment for Oreocharis fulvovillosa

## Additional information

### Artificial Intelligence (AI) use

The authors used AI-assisted language editing to improve the English expression of the manuscript.

### Author ORCIDs

Y. Wang https://orcid.org/0000-0002-2279-3828

H. Jiang https://orcid.org/0000-0001-6613-8588

B. Xu https://orcid.org/0000-0003-3507-9321

M.-Q. Han https://orcid.org/0000-0002-9059-7255

### Data availability

All of the data that support the findings of this study are available in the main text.
